# Raising the bar: genus-specific nested PCR improves detection and lineage identification of avian haemosporidian parasites

**DOI:** 10.3389/fcimb.2024.1385599

**Published:** 2024-04-29

**Authors:** Sandrine Musa, Theo Hemberle, Staffan Bensch, Vaidas Palinauskas, Laima Baltrūnaitė, Friederike Woog, Ute Mackenstedt

**Affiliations:** ^1^ Departement of Parasitology, University of Hohenheim, Stuttgart, Germany; ^2^ Department of Biology, Molecular Ecology and Evolution Lab (MEEL), Lund University, Lund, Sweden; ^3^ Nature Research Centre, Vilnius, Lithuania; ^4^ Departement of Ornithology, State Museum of Natural History Stuttgart, Stuttgart, Germany

**Keywords:** *Plasmodium*, *Haemoproteus*, *Leucocytozoon*, primers, mixed-infections

## Abstract

Avian haemosporidian parasites are useful model organisms to study the ecology and evolution of parasite-host interactions due to their global distribution and extensive biodiversity. Detection of these parasites has evolved from microscopic examination to PCR-based methods, with the mitochondrial cytochrome b gene serving as barcoding region. However, standard PCR protocols used for screening and identification purposes have limitations in detecting mixed infections and generating phylogenetically informative data due to short amplicon lengths. To address these issues, we developed a novel genus-specific nested PCR protocol targeting avian haemosporidian parasites. The protocol underwent rigorous testing utilizing a large dataset comprising blood samples from Malagasy birds of three distinct Passeriformes families. Furthermore, validation was done by examining smaller datasets in two other laboratories employing divergent master mixes and different bird species. Comparative analyses were conducted between the outcomes of the novel PCR protocol and those obtained through the widely used standard nested PCR method. The novel protocol enables specific identification of *Plasmodium*, *Haemoproteus* (*Parahaemoproteus*), and *Leucocytozoon* parasites. The analyses demonstrated comparable sensitivity to the standard nested PCR with notable improvements in detecting mixed infections. In addition, phylogenetic resolution is improved by amplification of longer fragments, leading to a better understanding of the haemosporidian biodiversity and evolution. Overall, the novel protocol represents a valuable addition to avian haemosporidian detection methodologies, facilitating comprehensive studies on parasite ecology, epidemiology, and evolution.

## 1 Introduction

The order Haemosporida, classified within the Apicomplexa, encompasses an important group of pathogens across all vertebrate groups. These unicellular blood parasites exhibit a heteroxenous life cycle, which requires alternation between vertebrate hosts and insect vectors (blood-sucking dipterans). In phylogenetic terms, avian Haemosporida of the genera *Plasmodium*, *Haemoproteus*, and *Leucocytozoon* are considered as the most ancient represantatives within their respective genera, signifying their ancestral status among vertebrate haemosporidian parasites (Pacheco et al., 2018a). Given their global distribution and substantial biodiversity (Clark et al., 2014), these genera are of scientific interest as model organisms to study the ecology and evolution of this parasite-host system (e.g. Bensch et al., 2000; Marzal et al., 2008; Pacheco et al., 2018a). Haemosporidian infections are often asymptomatic, can persist in the chronic phase within bird hosts over years (Valkiūnas, 2005), and the occurrence of mixed infections involving different genera or species of the same genus is common (Valkiūnas et al., 2006).

Before 2000, the detection of these parasites primarily relied on the microscopic examination of blood smears (Valkiūnas, 2005). However, with the emergence of molecular methods, PCR-based parasite detection gained popularity (Bensch et al., 2009). A fragment of the mitochondrial cytochrome *b* (cyt *b*) gene was identified as the target fragment that is now commonly used as a barcoding region of avian haemosporidian parasites. The identification is supported by the MalAvi database (Bensch et al., 2009), which contains data of over 5,000 unique avian haemosporidian lineages. The widely used detection method, a nested PCR targeting the barcoding region (Hellgren et al., 2004), is well-suited for simple screening due to its high sensitivity, although limitations have already been discovered in the detection of Haemosporida from Accipitridae (Harl et al., 2022, 2024). Additionally, there exist two further limitations: a short target fragment (479 bp), which limits its suitability for phylogenetic work, and insufficient detection of mixed infections with parasites of different genera (Bernotiene et al., 2016).

To address these limitations, alternative PCR protocols have been introduced. For instance, primer sets that enable amplifying longer gene fragments (Perkins and Schall, 2002; Schmid et al., 2017), or the entire mitochondrial genome (Pacheco et al., 2018a; Musa, 2023), are more suitable for phylogenetic studies. The inability of the standard detection method to adequately identify mixed infections is due to the primer pairs used. While there is a specific primer pair for *Leucocytozoon*, there is only one other pair that detects both *Plasmodium* and *Haemoproteus* parasites. In mixed infections, this often leads to one parasite genus being preferentially amplified due to the higher DNA concentration or more sensitive primer binding. Additional molecular methods, such as multiplex PCRs (Pacheco et al., 2018b; Ciloglu et al., 2019), have been introduced to uncover those mixed infections. Unfortunately, the target gene fragments of those methods cannot be used for lineage-level identification, as they only partially cover the barcoding region or not at all.

In response to these limitations, this study aims to devise a novel protocol using genus-specific primer sets for identifying avian haemosporidian parasites. This method is intended to ensure genus-specific amplification of the cyt *b* barcode, and still enable reliable identification of mixed infections between *Plasmodium* and *Haemoproteus* at the lineage level for the first time. The target fragment should also be larger than just the barcoding region to allow an improvement of phylogenetic analyses. Finally, the protocol should be designed in a way that is neither more complex nor cost-intensive compared to the standard method. The sensitivity and specificity of this new nested PCR assay, in comparison to the standard detection protocols, should be tested using different sample sets from different laboratories.

## 2 Materials and methods

### 2.1 Design of nested PCR primers

**Figure 1:**
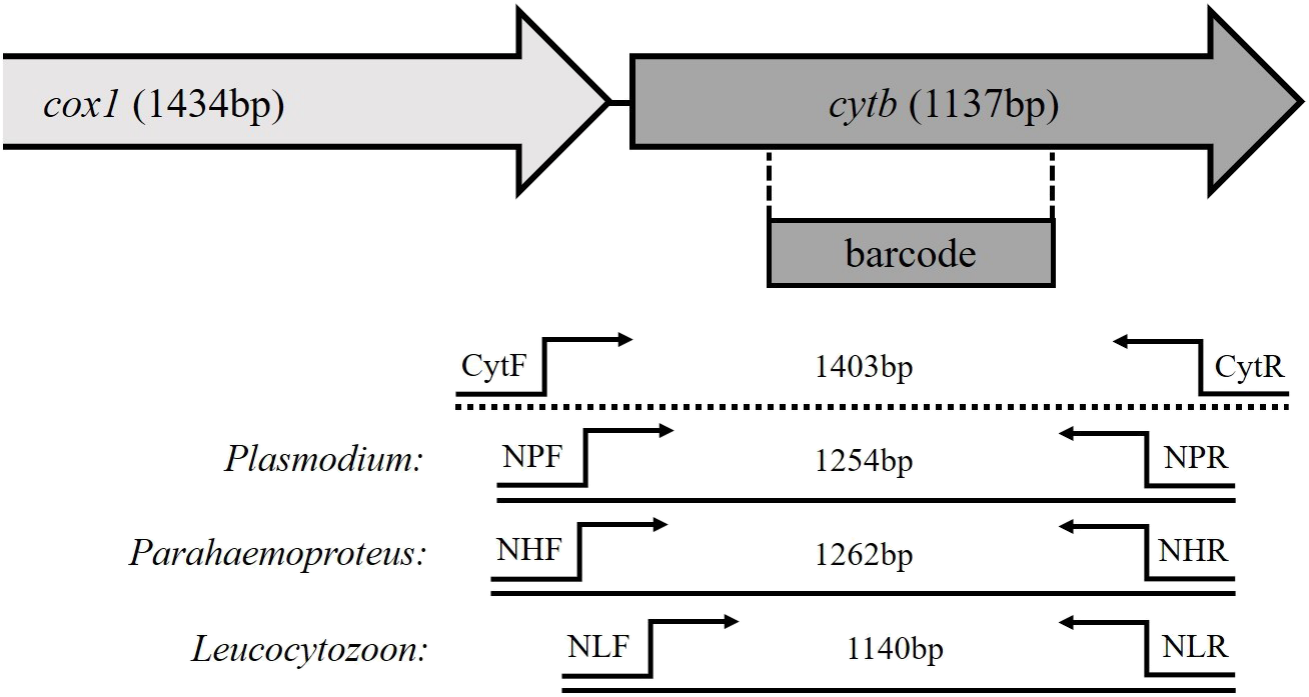
Schematic structure of partial mitochondrial genes of avian haemosporidian parasites. The location of the barcoding region is marked, as well as the primer binding sites of the newly developed genus-specific nested PCR and the length of their amplification products.

Using Geneious Prime 2023.2.1, a multiple alignment of 51 whole mitochondrial (mtDNA) genome sequences of avian *Plasmodium*, *Haemoproteus*, and *Leucocytozoon* isolates was prepared (list of accession numbers given in **Supplementary Table 1**). The sequences were derived from publications by Pacheco et al. (2018a) and Musa (2023). A manual search was performed for sequence motifs that occur in all genera and can be used as outer primers, as well as for motifs that are unique for each parasite genus to ensure genus-specific amplification in the nested PCR. The target fragment of all nested PCRs should be of comparable size and should contain the cyt *b* barcoding region. Since the subgenus *Haemoproteus* showed distinct sequence differences, different primer pairs were designed for the subgenera of *Haemoproteus*. Due to a lack of sample material for the evaluation of these primers, the primers of the subgenus *Haemoproteus* were not considered further in this study and only the primers for the subgenus *Parahaemoproteus* were evaluated. The binding sites of primers and sizes of the amplified fragments are shown in **Figure 1**.

### 2.2 Samples

The primary dataset of this study consisted of 264 blood samples of Malagasy bird species (**Table 1**) collected between 2003 and 2022 as part of an ongoing ringing project in the Maromizaha rainforest in eastern Madagascar.

**Table 1:** Primary dataset comprising 264 Malagasy bird blood samples from different avian taxa.

family	species	english name	N
Acrocephalidae			
	*Nesillas typica*	Madagascar Brush Warbler	84
	*Acrocephalus newtoni*	Madagascar Swamp Warbler	3
Ploceidae			
	*Ploceus nelicourvi*	Nelicourvi Weaver	63
Muscicapidae			
	*Copsychus albospecularis*	Madagascar Magpie Robin	57
	*Monticola sharpei*	Forest Rock-Thrush	5
	*Saxicola torquatus*	African Stonechat	52

Scientific names of family and species as well as the associated English name of the bird species and the total number of samples (N) are given.

The birds were caught in mist nets and a drop of blood was taken by puncturing the brachial vein. The protocol was approved by the Direction de la Préservation de la Biodiversité, Antananarivo, Madagascar. The blood was immediately stored in lysis buffer (Wink, 2006). Total DNA was extracted from the blood samples using the innuPREP Blood DNA Mini Kit (IST Innuscreen GmbH, Berlin, Germany) according to the manufacturer’s instructions. DNA concentration and purity were quantified using a NanoDrop N50 UV-Vis spectrophotometer (Implen GmbH, Munich, Germany) and samples were stored at –20°C until further use.

Two smaller datasets of naturally infected passerine birds were additionally tested, one at the Nature Research Centre, Vilnius, Lithuania and the other at the Molecular Ecology and Evolution Laboratory at Lund University in Lund, Sweden. The first dataset consisted of 54 blood samples collected at the Ventes Ragas ornithological station, Lithuania in 2019 – 2023 and the second of 52 samples collected at lake Krankesjön, Sweden in 2014-2017. For both these datasets, the blood was taken as described above from the brachial vein and stored in SET-buffer (0.05 M Tris, 0.15 M NaCl, 0.001 M EDTA, pH 8.0) (Hellgren et al., 2004). Total DNA extraction of blood samples was performed using an ammonium acetate protocol (Sambrook and Russel, 2001) or Quick-DNA Mini Prep (Zymo Research) DNA extraction kit and stored at –20°C until further use. The Swedish data set was previously analysed within a large host-parasite community study (Ellis et al., 2020) and because the extracted samples had been exposed to repeated thawing and freezing, the DNA might have become degraded. Therefore, DNA was re-extracted from ten samples that failed parasite detection in the first round of analyses.

### 2.3 Standard nested PCR protocol

To evaluate the sensitivity and specificity of the new nested PCR assay in comparison to the broadly used standard diagnostic method, the blood samples of the Malagasy birds were at first screened for haemosporidian parasites using the following protocol. Each sample was separately tested for the presence of *Plasmodium* spp./*Haemoproteus* spp. using the primers HAEMNF/HAEMNR2 (Waldenström et al., 2004) and HAEMF/HAEMR2 (Bensch et al., 2000) and *Leucocytozoon* spp. using the specific primers HAEMNF/HAEMNR3 and HAEMFL/HAEMR2L (Hellgren et al., 2004). Reaction mixtures of the separate nested PCRs were identical. PCR reactions of the first run were carried out in a total volume of 25 μl containing 2.5 μl10X ReproFast Buffer with 20mM MgSO_4_ (Genaxxon bioscience GmbH, Ulm, Germany), 1 μl of each primer (HAEMNF/HAEMNR2 or HAEMNF/HAEMNR3; 10 mM), 0.5 μl of each dNTP (10 μmol), 0.125 μl ReproFast-DNA Polymerase (proofreading; 5 U/μl; Genaxxon bioscience GmbH, Ulm, Germany), 5 μl template DNA (10–100 ng/μl) and 14.875 μl nuclease-free water. The PCR amplification protocol started with an initial denaturation step of 94°C for 5 min, followed by 20 cycles of denaturation at 94°C for 30 s, annealing at 50°C for 30 s and extension at 72°C for 45 s. The final extension takes place at 72°C for 5 min. The reaction mixture of the nested PCRs consisted of 5 μl10X ReproFast Buffer with 20mM MgSO_4_ (Genaxxon bioscience GmbH, Ulm, Germany), 2 μl of each primer (HAEMF/HAEMR2 or HAEMFL/HAEMR2L; 10 mM), 1 μl of each dNTP (10 μmol), 0.25 µl ReproFast-DNA Polymerase (proofreading; 5 U/μl; Genaxxon bioscience GmbH, Ulm, Germany), 2 μl amplification product of the initial PCR and 37.75 μl nuclease-free water in a total volume of 50 μl. The cycling conditions included an initial denaturation step of 94°C for 5 min, followed by 35 cycles of denaturation at 94°C for 30 s, annealing at 50°C (HAEMFL/HAEMR2L) or 55°C (HAEMF/HAEMR2) for 30 s and extension at 72°C for 45 s. The final extension was carried out at 72°C for 5 min.

Lithuanian samples were tested with the same primer sets as Malagasy birds using conditions described by Bensch et al. (2000) and Hellgren et al. (2004). PCR reactions were carried out in a total volume of 25 μl, in the first reaction containing 12.5 μl Dream Taq Green Master Mix (2X) (Thermo Fischer Scientific, Lithuania), 8.5 μl of ultra-pure water, 1 μl of each primer, and 2 μl of template DNA. 2 μl of the first reaction product was used as a DNA template in the second reaction.

The Swedish samples had previously been analysed (Ellis et al., 2020) following the same procedure as for the Lithuanian samples with the only difference in the use of DNA polymerase (AmpliTaq^©^ DNA Polymerase, applied biosystems). Before testing the 52 samples with the GS-protocol, the samples were screened (in September 2023) using a multiplex PCR assay (Ciloglu et al., 2019) to verify the infections recorded by Ellis et al. (2020).

### 2.4 Genus-specific nested PCR protocol

The newly established genus-specific nested PCR was performed running an unspecific PCR in the first place using the primers CytF (5´-GGARCAATAATTGSATTATTTAC-3´) and CytR (5´-TGCTTGDGAGCTGTAATCAT-3´) amplifying a fragment of 1,403bp, following by three separate, genus-specific nested PCRs using the amplification product of the first PCR as template. The primer pair NPF (5´-CGTGAAAAYTCAATAATAATAYTATGGTC-3´)/NPR (5´-AACGACCATATAAAATGTAAATATC-3´) was used for the detection of *Plasmodium* (1,254bp), NHF (5´-TAAACATTTACGTGATAATACAATAATTG-3´)/NHR (5´-CGACCATATAAAATGAAAATAGA-3´) for *Haemoproteus* (*Parahaemoproteus*; 1,262bp) and the primers NLF (5´-GACGTATACCTGATTATCCTGATAA-3´)/NLR (5´-TTTGTGGTAATTGATAACCTATAMMCAT-3´) for *Leucocytozoon* detection (1,1140bp). The reaction mixtures of the different PCRs were similar to those of the standard nested PCR protocol described above. Cycling conditions of the different PCRs are given in **Table 2**. Reaction mixtures of the PCRs for Lithuanian samples were using Dream Taq Green (2X) Master Mix (Thermo Fischer Scientific, Lithuania) as with standard protocol and following thermal conditions indicated in **Table 2**.

**Table 2:** Cycle conditions of newly established genus-specific nested PCR. First PCR was performed using primer pair CytF/CytR, whereas nested PCRs use genus-specific primer pairs (NPF/NPR for *Plasmodium* spp., NHF/NHR for *Haemoproteus* (*Parahaemoproteus*) spp. and NLF/NLR for *Leucocytozoon* spp.).

primer pair	initial denaturation 5 min	denaturation 30 s	annealing 30 s	extension 80 s	final extension 5 min	cycles
*CytF/CytR*	94° C	94° C	45° C	72° C	72° C	25
*NPF/NPR*			55° C			35
*NHF/NHR*			50° C			
*NLF/NLR*			55° C			

The number of cycles implementing denaturation, annealing and extension differs between the first PCR (25 cycles) and nested PCRs (35 cycles).

**Figure 2:**
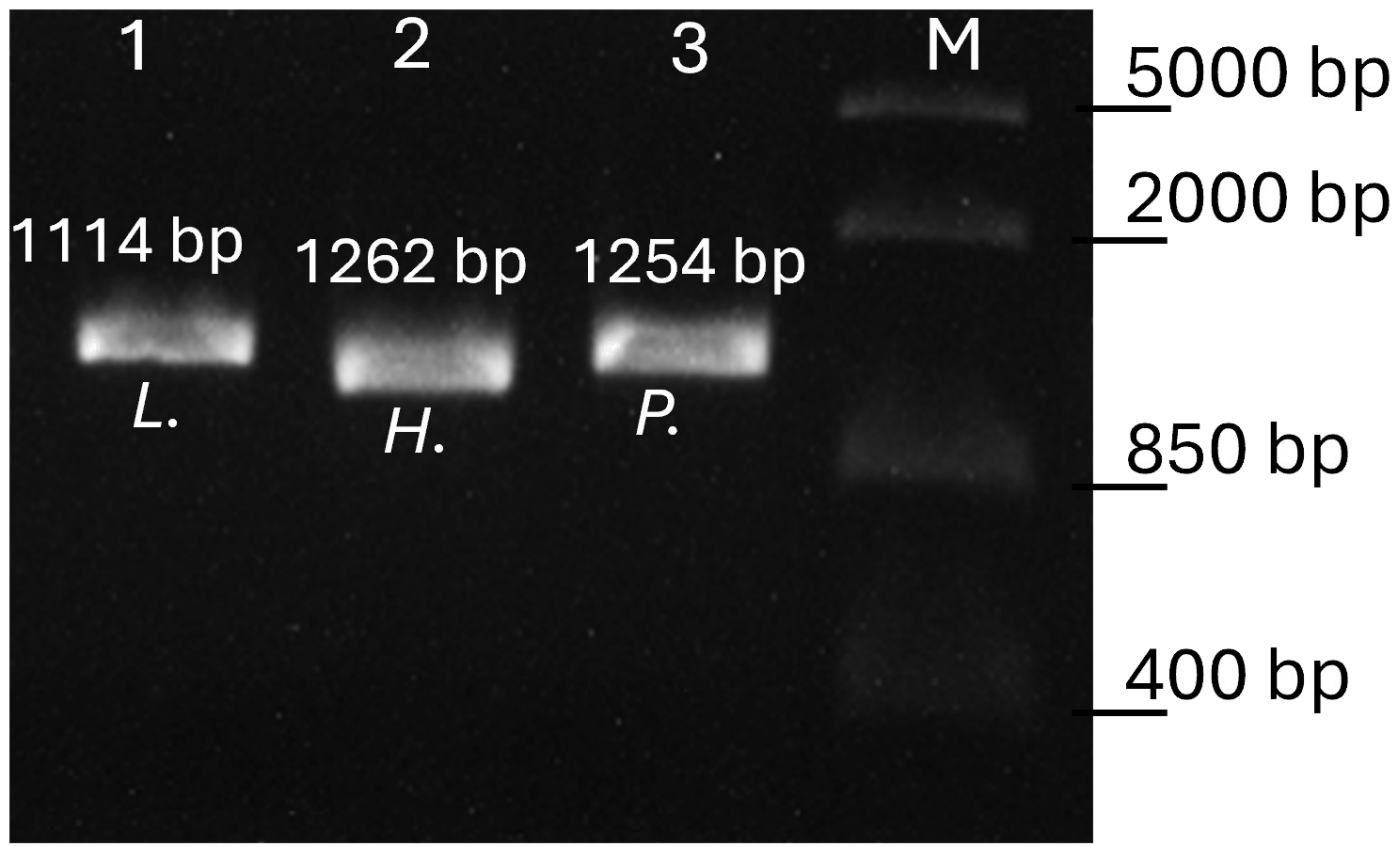
Agarose gel electrophoresis of the genus-specific PCR products. Lane 1 amplicon of *Leucocytozoon (L.)*, lane 2 amplicon of subgenus *Parahaemoproteus (H.)* and lane 3 *Plasmodium (P.)* infection. Lane M, FastRuler Middle Range DNA Ladder (Thermo Scientific). Fragment sizes including primers are given.

The amplification products (5 μl) of all nested PCR assays were mixed with GelRed™ stain and a loading buffer and then visualized on a 1.5% agarose gel after 20 min at 90 V. Amplification products (**Figure 2**) were then purified using the PCR Product Purification Kit (Roche, Mannheim, Germany) and after sequencing (Microsynth AG, Switzerland), the resulting sequence data were edited using Geneious v. 2021.1.1. The final sequences were then distinguished by identifying their closest match using a BLAST search of the MalAvi database (Bensch et al., 2009); as of November 2023. All newly detected lineages were deposited in GenBank (accession numbers OR901950-OR901956). To minimize false-negative results, the PCR was repeated for each sample that showed either no band in the agarose gel or insufficient sequencing results.

### 2.5 Phylogenetic analysis

To test for differences in phylogenetic analyses, due to the longer fragment size gained from the genus-specific nested PCR, two different phylogenetic analyses were performed. The first consisted of avian haemosporidian lineages from the standard nested PCR, each trimmed to the length of the barcoding region, 479 bp. The second dataset included sequences of the same lineages, amplified by the genus-specific nested PCR, each trimmed to a homologous fragment of 1,000 bp. Both datasets were phylogenetically analysed using MEGA v.10.2 (Kumar et al., 2018). Both analyses were performed running the general time reversible model with gamma distribution (GTR + G). All maximum likelihood methods were performed using 1000 replicates. The resulting phylograms were viewed and edited with MEGA v.10.2.

### 2.6 Statistical analysis

To compare the sensitivity of the genus-specific nested PCR method described here to that of the typical nested PCR protocol, both methods were used to assess the infection status of the Malagasy individual bird blood samples and the parasites lineage identity. The prevalence by each parasite genus and that of mixed infections was compared between the two methods using chi-squared tests. The number of additional positives in the second test run of each method was compared to elucidate the repeatability of results. Each detected haemosporidian parasite lineage was classified as either: a) detected in both methods, b) detected exclusively in this method, c) lineage of the same genus, but different lineage or d) lineage not identified due to sequencing errors. Those categories were compared between the two methods. Results of the sample sets from Lithuania and Sweden were separately analysed by comparing prevalences using chi-squared tests. Statistical analysis was performed using DATAtab.

## 3 Results

The initial dataset, comprising 264 Malagasy bird blood samples, disclosed a haemosporidian parasite prevalence of 58.7% through the SN protocol, whereas the recently introduced GS protocol yielded a prevalence of 50.75%. The observed variance in detection probabilities did not attain statistical significance (χ2 = 3.371, df = 1, p = 0.066). The prevalence of *Haemoproteus* exhibited similar values between both methods (SN: 17.4%, GS: 18.2%), while *Plasmodium* was more frequently identified using the SN protocol (SN: 37.5%, GS: 31.1%), and *Leucocytozoon* was more prevalent with the GS protocol (SN: 25%, GS: 32.2%). Upon retesting negative samples, the genus-specific protocol demonstrated a lower incidence of false-negatives in the initial round, as illustrated in **Table 3**.

**Table 3:** Comparison of the number of positive tested samples (n) between the standard nested PCR approach (SN) and the genus-specific nested PCR (GS).

method	*Plasmodium*		*Haemoproteus*		*Leucocytozoon*	
	Round 1	Round 2	Round 1	Round 2	Round 1	Round 2
SN	83	+17	34	+13	49	+19
GS	80	+5	47	+6	82	+4

The table displays the count of positive samples identified in the initial round of testing and the supplementary positives (n) from the second round, categorized by haemosporidian genus.

The genus-specific nested PCR identified a higher number of mixed infections compared to the standard nested PCR, although this difference is not statistically significant (χ2 = 1.255, df = 1, p = 0.263). Using the SN protocol, 58 double infections were identified, consisting of 43 *Plasmodium/Leucocytozoon* and 15 *Haemoproteus/Leucocytozoon* mixed infections. In addition, 31 samples contained sequences showing double peaks, suggesting mixed infections with species/haplotypes of the same genus. In contrast, the GS protocol successfully detected 55 double infections, including 39 *Plasmodium/Leucocytozoon*, 8 *Haemoproteus/Leucocytozoon*, and 8 *Plasmodium/Haemoproteus* mixed infections. Additionally, the GS protocol identified 14 samples containing DNA from all three haemosporidian genera. The sequences of 29 samples showed double peaks, indicating mixed infections of species/haplotypes of the same genus.

The combination of both methods revealed with 80.9% the highest prevalence for Ploceidae, namely *Ploceus nelicourvi*, and the lowest prevalence for Muscicapidae with 50%. Acrocephalidae showed a prevalence of 66.7% for haemosporidian parasite infections (**Table 4**). All avian species exhibited mixed infections, with a notable prevalence of co-infections involving both *Plasmodium* and *Leucocytozoon* spp. within samples of *Ploceus nelicourvi.*

**Table 4:** Haemosporidian infections of Malagasy bird species.

family	species	N	prevalence	lineages (n)
Acrocephalidae			**66.7**	
	*Nesillas typica*	84	66.7	pFOUMAD03 (16), pGRW04 (4), pGRW09 (3), hACNEW01 (3), hNESTYP01 (13), hNESTYP02, hNEWBR03, hURANO02 (3), lCINSOV02, lFOMAD01 (19), lNESTYP03 (3), lPLONEL02, lZOMAD01
	*Acrocephalus newtoni*	3	66.7	hACNEW02, hNESTYP01, lFOMAD01
Ploceidae			**80.9**	
	*Ploceus nelicourvi*	63	80.9	pCOLL7, pFOUMAD03, pFOUOMI04, pGRW04 (24), pGRW09 (12), pLINOLI01, hACNEW01, hNESTYP01, hPLONEL01 (3), hPLOSAK01 (2), hRBQ01, hSFC3 (2), lFOMAD01 (8), llHYPMA02, lPLONEL02 (14), lPLONEL03, lPLONEL04, lPLONEL05 (2), lPLONEL06, lPHICAS01
Muscicapidae			**50**	
	*Copsychus albospecularis*	57	56.1	pCOPALB03 (2), pFOUMAD03, pGRW04 (15), pNEWAM07, pSATOR01, pSATOR02, pWW3, hSFC3 (15), lFOMAD01 (10), lRECOB3
	*Monticola sharpei*	5	60	pGRW04 (3), pFOUMAD03, pGRW09, hMONSHA01, lFOMAD01
	*Saxicola torquatus*	52	42.3	pFOUMAD03 (2), pGRW04 (7), pGRW09 (9), pSATOR02, hSFC3 (2), lFOMAD01, lRECOB3 (3)

Total number of examined samples (N), prevalence and identified haemosporidian lineages are given. Number of lineage detections are written if they exceed 1. Bold text indicates the prevalence of the bird family.

Sequencing revealed 35 distinct haemosporidian lineages in the Malagasy sample set, encompassing 11 *Plasmodium*, 11 *Haemoproteus*, and 13 *Leucocytozoon* lineages. Notably, *Ploceus nelicourvi* exhibited the highest diversity, hosting a total of 20 different lineages, followed by *Nesillas typica* with 13 different lineages (**Table 4**). Four lineages (pFOUMAD03, pGRW04, pGRW09 and lFOMAD01) were detected in all three avian bird families, whilst two *Haemoproteus* lineages (hACNEW01 and hNESTYP01) were shared between Acrocephalidae and *Ploceus nelicourvi* and hSFC3 was found in *Ploceus nelicourvi* as well as in Muscicapidae. The detection rate of some haemosporidian lineages differed notably between the two nested PCR protocols. The standard nested PCR revealed more infections with pGRW09 and hNESTYP01, while the genus-specific PCR was able to detect more infections of hSFC3 and lFOMAD01 (**Table 5**).

**Table 5:** Number of haemosporidian lineage detections within the Malagasy sample set using standard nested PCR protocol (SN) and genus-specific nested PCR protocol (GS).

genus	lineage name	Acc. No.	N (SN)	N (GS)
*Plasmodium*	pGRW04	AF254975	43	41
	pGRW09	DQ060773	25	10
	pFOUMAD03	JN661983	15	16
	pHYPMA01	JN661998	3	2
	pCOPALB03	MF442560	2	0
	pSATOR02	MF442564	2	2
	pCOLL7	DQ368376	1	1
	pFOUOMI04	MF442548	1	1
	pLINOLI01	DQ659554	0	1
	pNEWAM07	MF442549	0	1
	pWW3	AF495577	0	1
*Haemoproteus*	hNESTYP01	MF442572	15	7
	hSFC3	DQ060771	11	17
	hACNEW01	KX506762	4	2
	hURANO02	MF442579	3	3
	hACNEW02	MF442594	1	0
	hNESTYP02	MF442582	1	1
	hNEWBR03	MF442596	1	0
	hPLOSAK01	JN661931	1	2
	hMONSHA01	MF442586	0	1
	hPLONEL01	MF442606	0	3
	hRBQ01	AF495567	0	1
*Leucocytozoon*	lFOMAD01	JN032605	26	39
	**lPLONEL02**	OR901951	8	14
	lRECOB3	DQ847221	4	3
	**lNESTYP03**	OR901950	2	3
	**lPLONEL04**	OR901953	1	1
	lHYPMA02	MF442609	1	1
	lZOMAD01	JN032614	1	0
	lCINSOV02	OR347658	0	1
	**lPLONEL03**	OR901952	0	1
	**lPLONEL05**	OR901954	0	2
	**lPLONEL06**	OR901955	0	1
	lPHICAS01	OR347663	0	1
	**lPLONEL07**	OR901956	1	0

Genus, name, and Accession number of each lineage are given. Names of newly identified lineages are written in bold.

**Figure 3:**
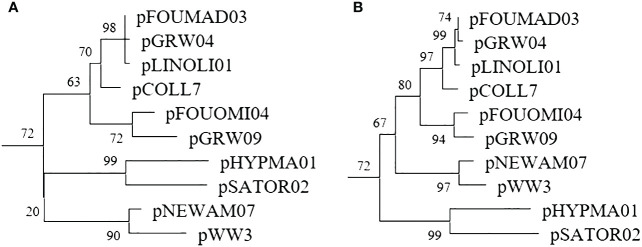
*Plasmodium* subtree of the phylogenetic trees constructed with a 479bp fragment amplified by the standard nested PCR **(A)** and a 1,000bp fragment amplified by the genus-specific nested PCR **(B)**.

Phylogenetic examination of the larger fragment amplified by the genus-specific nested PCR resulted in an overall better-defined phylogenetic topology of lineages (**Figure 3**) and higher bootstrap values (**Supplementary Material**).

The analysis of the second dataset, encompassing 54 bird blood samples examined at the Nature Research Centre in Vilnius, unveiled marginally reduced detection rates for all haemosporidian genera when utilizing the genus-specific nested PCR method (**Table 6**). However, none of these differences reached statistical significance. Notably, with respect to *Plasmodium* detections, the genus-specific nested PCR exhibited a failure to identify three samples that had tested positive using the standard nested PCR. Conversely, this method successfully amplified two additional positive samples. Subsequent sequencing of these samples resulted in the identification of *Plasmodium circumflexum* (pTURDUS1) within this subset of samples.

**Table 6:** Haemosporidian parasite infections detected in the Lithuanian sample set (N = 54) using standard nested PCR protocol (SN) and genus-specific nested PCR protocol (GS).

method	*Plasmodium*	*Haemoproteus*	*Leucocytozoon*
SN	18	18	22
GS	17	14	19

The third dataset, examined at the Molecular Ecology and Evolution Laboratory at Lund University, comprised 52 bird blood samples from passerines. The sample set was previously tested with the multiplex PCR assay and standard nested PCR. The success rate was ~50% lower for the GS protocol compared to the other two protocols (**Table 7**). The GS protocol detected one additional *Haemoproteus* infection, that was identified as *H. fringillae* hCCF3 (DQ060764).

**Table 7:** Haemosporidian parasite infections detected in the Swedish sample set (N = 52) using multiplex PCR (MP), standard nested PCR protocol (SN) and genus-specific nested PCR protocol (GS).

method	*Plasmodium*	*Haemoproteus*	*Leucocytozoon*
MP	28	26	20
SN	27	21	20
GS	14(+5)	12(+4)	11(+4)

Additional positives of re-extracted samples (n=10) using the GS protocol are written in brackets.

To test whether this was due to degraded DNA, we tested the GS protocol again on re-extracted DNA from ten samples that failed parasite detection in the first test round. These ten samples had been scored to contain a total of 16 infections (6 single, 2 double and 2 triple infections) by the MP and SN protocols. In this second test with the GS protocol, we recovered 13 of these 16 infections.

## 4 Discussion

In this study, novel genus-specific primers were designed to facilitate the specific identification of avian haemosporidian parasites belonging to the genera *Plasmodium, Leucocytozoon*, and the subgenus *Parahaemoproteus*. The newly developed protocol underwent rigorous testing utilizing a large dataset comprising blood samples from Malagasy birds of three distinct Passeriformes families. Furthermore, validation occurred through application to smaller datasets in two other laboratories employing divergent master mixes and different bird species. Comparative analyses were conducted between the outcomes of the novel PCR protocol and those obtained through the widely used standard nested PCR method. Notably, the latter was either run as an integral component of this investigation or had been previously carried out in other research projects.

The comprehensive evaluation of the Malagasy and Lithuanian datasets yielded a sensitivity comparable to, albeit slightly lower than, that of the standard nested PCR, although those differences were not statistically significant. In contrast, the Swedish dataset exhibited only a 50% success rate in detecting all known haemosporidian infections. It was hypothesized that this lower success rate might be attributable to the slight degradation of DNA within the samples, likely resulting from prolonged use. Degraded DNA is anticipated to have a more pronounced impact on the GS protocol compared to the SN and MP protocol due to the considerably longer amplicons (>1000 bp versus <500 bp). Subsequent analyses involving the extraction of freshly obtained DNA from samples that initially failed amplification yielded a recovery of the majority of the missed infections (81.25%). This highlights the importance of DNA quality in facilitating the amplification and sequencing especially of longer fragments (Freed and Cann, 2006), thus ensuring the reliability and comparability of results obtained through the GS protocol. To confirm the integrity of DNA samples, it is advisable to initially assess the total DNA quantity in the extracted samples, either through photometry or by examining the extractions on agarose gels (Reinoso-Pérez et al., 2020). In order to ensure a baseline level of DNA quality, it may be beneficial to conduct molecular amplification of host DNA simultaneously. However, it is important to note that this approach might not completely eliminate the possibility of moderate levels of DNA degradation which could affect the quality of haemosporidian DNA. Subtle variations in sensitivity are likely attributable to the primer specificity. Within the Malagasy dataset, the standard nested PCR demonstrated a notably higher detection rate for pGRW09, while the genus-specific nested PCR exhibited a greater frequency in detecting lFOMAD01. As the complete mitochondrial DNA (mtDNA) of lFOMAD01 (OR347660) is available, an analysis of primer binding sites was feasible. When considering the primers utilized in the GS protocol, only one mismatch is observed for the primer CytR. On the other hand, the primers employed in the NS protocol show three mismatches (2 for HaemNF and 1 with HaemNR2L). The primers used in these protocols demonstrate distinct affinities to the sequence of the respective lineage, leading to noticeable variations in prevalence. In the instance of *Haemoproteus* (*Parahaemoproteus*), where both protocols demonstrate a comparable prevalence, individual distinctions persist in the detection rates of specific lineages. For instance, hNESTYP01 was more frequently identified through the standard nested PCR, contrasting with the preference of the genus-specific nested PCR for detecting hSFC3. Given the great diversity among avian Haemosporida, the design of primers that are specific for all lineages within a genus presents an enormous challenge, if not an impossible one. The documented existence of diverse primer preferences in other protocols (Bernotiene et al., 2016) substantiates these findings. To comprehensively capture most lineages in mixed infections, the simultaneous application of at least three PCRs is recommended. Due to the significant effort needed in applying various PCR approaches, this recommendation has not been heeded. The standard nested PCR method remains the method of choice in the most recent studies conducted (e.g. Žiegytė et al., 2023; González-Olvera et al., 2023; Han et al., 2023). It was only complemented by the utilization of multiplex PCRs (Pacheco et al., 2018b; Ciloglu et al., 2019) in some studies (e.g. Musa et al., 2022; Barbon et al., 2023; Procházka et al., 2023) due to its limitation in detecting mixed infections of *Plasmodium* and *Haemoproteus*. However, those assays have failed to ensure a definitive identification at the parasite’s lineage level. The protocol presented in this study enables this identification for the first time by amplifying fragments that overlap with the haemosporidian barcoding region (Bensch et al., 2009). This allows for a thorough investigation into the exact composition of mixed infections. Furthermore, in the case of sequences exhibiting double peaks, usually only the genus can be determined, and it is assumed that a mixed infection of two lineages/species of the same genus is present. However, with the SN protocol, one cannot be entirely certain whether the double peaks detected in sequences resulting from the use of the primer pair HaemF/HaemR2 also represent a mixed infection of *Plasmodium* and *Haemoproteus* lineages. The use of the GS protocol ensures a more reliable identification of these double infections. Nevertheless, it is important to keep in mind that the observed frequency of mixed infections, prevalence, or biodiversity of haemosporidian parasites in a population is consistently underestimated (Valkiūnas et al., 2006).

Furthermore, it must be noted that the dataset of blood samples examined in this study, comes from Passeriformes. Haemosporidian parasites from other bird families show sometimes extreme differences in their mitochondrial genome. For example, the barcoding region of *Haemoproteus elani* hBUBT1, which infects Accipitriformes, exhibits only an 87% homology to other avian Haemosporida (Harl et al., 2022). Therefore, it cannot be ruled out that, in addition to the subgenus *Haemoproteus*, other haemosporidian lineages from birds of different bird families may not be detected by the primers developed here. Further studies should therefore investigate the detection rates more precisely with datasets from non-Passeriformes.

Methods for detecting avian haemosporidians can be categorized based on their primary purpose, such as screening, identification, or phylogeny (Bensch and Hellgren, 2020). Phylogenetic analyses are particularly crucial for investigating evolutionary factors, and the accuracy of such analyses relies heavily on the length of DNA fragments. Previous research has demonstrated that, for robust phylogenetic analyses, the barcoding region (479 bp) is insufficient for obtaining well-supported topologies, with only a few nodes showing bootstrap values >70% (Ellis and Bensch, 2018). When comparing phylogenetic trees constructed from the lineages detected in the Malagasy sample set, it was observed that 26.9% of nodes had bootstrap values <70% when using the barcoding region, whereas this decreased to 18.5% when using the 1,000 bp long fragment amplified by the genus-specific nested PCR. The utilization of these longer fragments enhances the resolution of the phylogenetic topology of lineages.

In conclusion, the newly described genus-specific nested PCR protocol addresses a crucial gap in the avian haemosporidian detection toolkit. It facilitates lineage-specific identification of *Plasmodium*, *Haemoproteus* (*Parahaemoproteus*), and *Leucocytozoon* parasites. The effectiveness of this innovative protocol has been demonstrated across various laboratories, yielding comparable results if DNA extract was of good quality. It utilizes the same equipment and requires nearly the same amount of time compared to the standard nested PCR, yet eliminates an additional step in identifying mixed infections of *Plasmodium* and *Haemoproteus*. Moreover, the length of the amplification product enhances the robustness of phylogenetic analyses. This holds significant importance for molecular epidemiological surveillance and biodiversity studies, enabling a thorough examination of mixed infections.

## Data availability statement

The datasets presented in this study can be found in online repositories. The names of the repository/repositories and accession number(s) can be found in the article/**Supplementary Material**.

## Ethics statement

Ethical approval was not required for the study involving animals in accordance with the local legislation and institutional requirements because Sample material was obtained in the course of other studies.

## Author contributions

SM: Conceptualization, Methodology, Writing – original draft, Writing – review & editing. TH: Conceptualization, Investigation, Writing – review & editing. SB: Investigation, Writing – review & editing. VP: Writing – review & editing. LB: Investigation, Writing – review & editing. FW: Writing – review & editing. UM: Writing – review & editing.
