# Supplementary material for: Raising the bar: genus-specific nested PCR improves detection and lineage identification of avian haemosporidian parasites
Source: Front Cell Infect Microbiol. 2024 Apr 29;14:1385599. doi: 10.3389/fcimb.2024.1385599 (PMC11089177; doi:10.3389/fcimb.2024.1385599)
Supplement: Supplementary file 1 [file DataSheet_1.docx]

Supplementary Material

# Supplementary Data

**Supplementary Table 1.** Haemosporidian lineages used for building of multiple alignment.

| lineage name | Morphospecies | Accession number |
| --- | --- | --- |
| hROBIN1 | *Haemoproteus balmorali* | KY653800 |
| hHIICT1 | *Haemoproteus belopolskyi* | KY653790 |
| hTANIG01 | *Haemoproteus coatneyi* | KY653752 |
| hHAECOL1 | *Haemoproteus columbae* | KY653761 |
| hZOCAP14 | *Haemoproteus erythrogravidus* | KY653797 |
| hCREFUR01 | *Haemoproteus jenniae* | KY653758 |
| hRB1 | *Haemoproteus lanii* | KY653787 |
| hRBQ11 | *Haemoproteus micronuclearis* | OR327004 |
| hTURDUS2 | *Haemoproteus minutus* | KY653763 |
| hYWT3 | *Haemoproteus motacillae* | KY653768 |
| hLAMPUR01 | *Haemoproteus pastoris* | KY653793 |
| hBUL2 | *Haemoproteus sanguinis* | OR327000 |
| hSISKIN1 | *Haemoproteus tartakovskyi* | KY653809 |
| hTROAED20 | *Haemoproteus wittii* | KY653794 |
| lGALLUS05 | *Leucocytozoon caulleryi* | AB302215 |
| lASOT3 | *Leucocytozoon danilewskyi* | KY653781 |
| lTUMER09 | *Leucocytozoon dubreuili* | KY653795 |
| lTRPIP2 | *Leucocytozoon fringillinarum* | KY653765 |
| lZOLEU02 | *Leucocytozoon majoris* | FJ168563 |
| lHEAME01 | *Leucocytozoon quynzae* | KF479480 |
| lGALLUS08 | *Leucocytozoon sabrazesi* | AB299369 |
| pGRW02 | *Plasmodium ashfordi* | KY653775 |
| pTURDUS1 | *Plasmodium circumflexum* | KY653762 |
| pGRW06 | *Plasmodium elongatum* | KY653801 |
| pCOLL4 | *Plasmodium homocircumflexum* | KY653784 |
| pBAEBIC02 | *Plasmodium homopolare* | KY653770 |
| pDIGLAF01 | *Plasmodium lutzi* | KY653815 |
| pSGS1 | *Plasmodium relictum* | KY653774 |
| pGRW04 | *Plasmodium relictum* | OR347671 |
| pTFUS06 | *Plasmodium unalis* | KY653814 |
| pSYAT05 | *Plasmodium vaughani* | KY653792 |
| hNEWBR04 | unknown | OR327002 |
| hFOUMAD02 | unknown | OR326999 |
| hNEWAM04 | unknown | OR327001 |
| hNEWBR05 | unknown | OR327003 |
| pBUL07 | unknown | OR347666 |
| pCOPALB03 | unknown | OR347669 |
| pNEWAM05 | unknown | OR347673 |
| pHYPMA01 | unknown | OR347672 |
| pWW3 | unknown | OR347675 |
| pCOLL7 | unknown | OR347668 |
| pNEWAM07 | unknown | OR347674 |
| pFOUMAD03 | unknown | OR347670 |
| lHYPMA02 | unknown | OR347661 |
| lFOMAD01 | unknown | OR347660 |
| lHYPMA03 | unknown | OR347662 |
| lPICPIC03 | unknown | OR347665 |
| lPICPIC01 | unknown | OR347664 |
| lCOCOR09 | unknown | OR347659 |
| lPHICAS01 | unknown | OR347663 |
| lCINSOV02 | unknown | OR347658 |

**
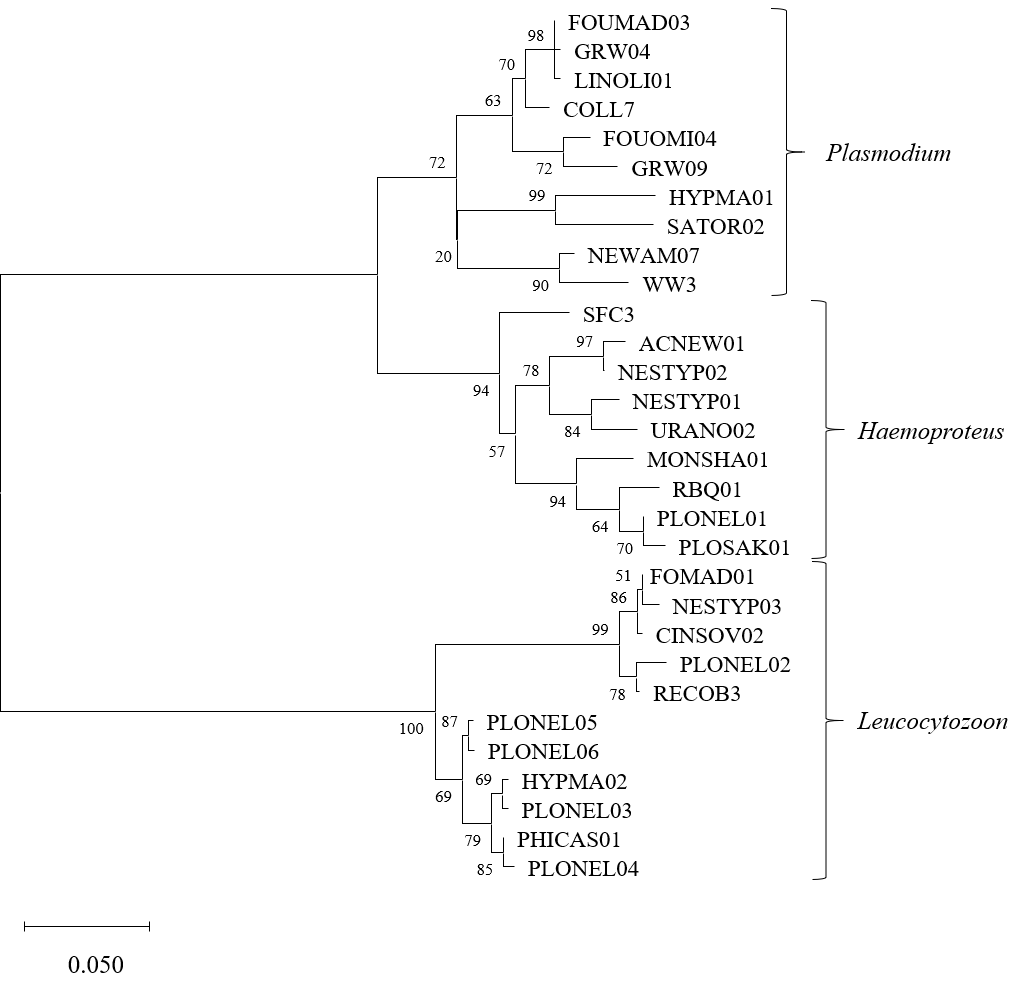
**

**Supplementary Figure 1.** Phylogenetic tree of haemosporidian lineages detected in the Malagasy sample set using the standard nested PCR. The analysis was performed using a 479bp fragment of the cytochrome *b* gene, running the general time reversible model with gamma distribution (GTR + G) with 1000 replicates in MEGA v.10.2 (Kumar *et al.*, 2018).


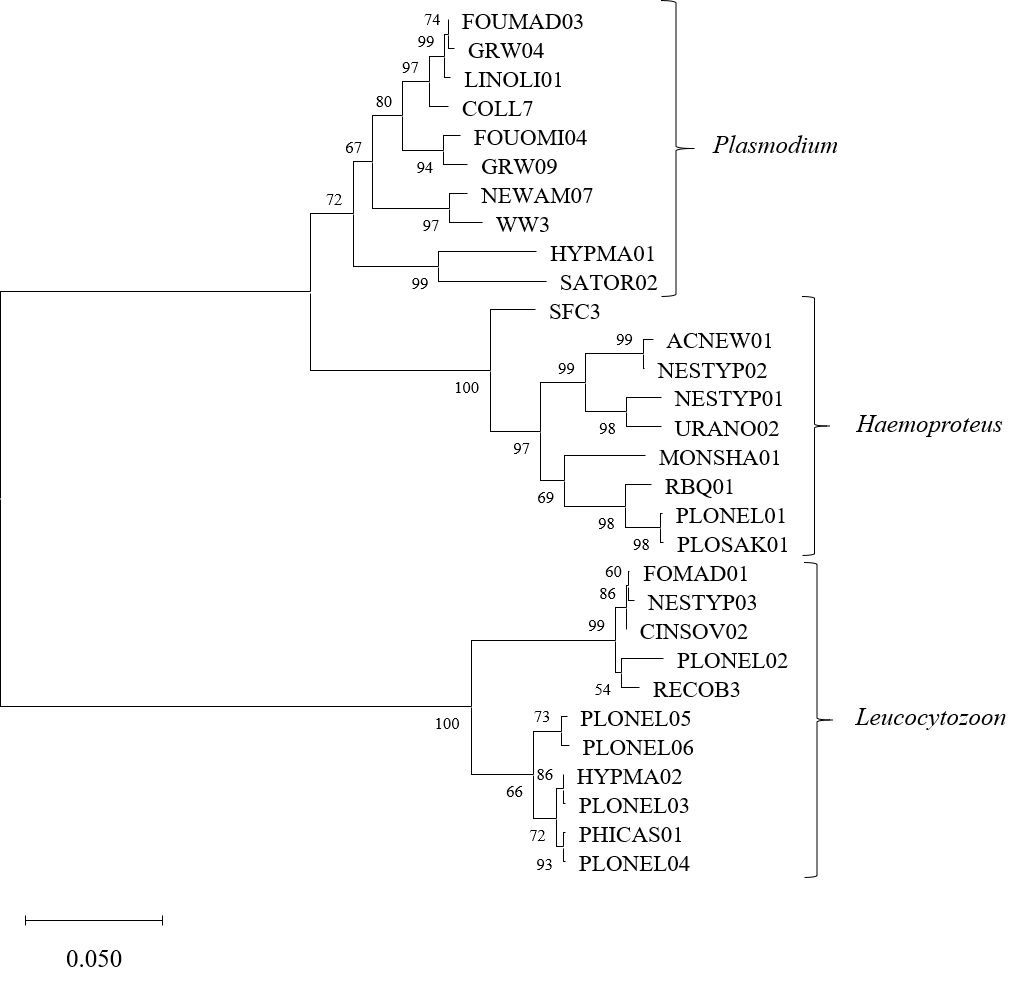


**Supplementary Figure 2.** Phylogenetic tree of haemosporidian lineages detected in the Malagasy sample set using the genus-specific nested PCR. The analysis was performed using a 1,000bp fragment of the cytochrome *b* gene, running the general time reversible model with gamma distribution (GTR + G) with 1000 replicates in MEGA v.10.2 (Kumar *et al.*, 2018).
